# Strength Enhancement of Laser Powder Bed Fusion 316L by Addition of Nano TiC Particles

**DOI:** 10.3390/ma17051129

**Published:** 2024-02-29

**Authors:** Yanyan Liu, Deqiao Xie, Fei Lv

**Affiliations:** 1 College of Intelligent Manufacturing, Nanjing University of Science and Technology Zijin College, Nanjing 210046, China; 2 College of Astronautics, Nanjing University of Aeronautics and Astronautics, Nanjing 210016, China; dqxie@nuaa.edu.cn; 3 Shanghai Key Laboratory of All Solid-State Laser and Applied Techniques, Shanghai Institute of Optics and Fine Mechanics, Chinese Academy of Sciences, Shanghai 201800, China

**Keywords:** 316L, laser powder bed fusion, TiC, mechanical property, high temperature property

## Abstract

316L stainless steel is widely used in various industrial fields, but its strength is relatively low. The improvement of its strength has become a research hotspot. In this study, nano titanium carbide (TiC) particles are ball milled with 316L with the addition of 2 wt% and 4 wt%. The composite powder was then used for the fabrication of samples by laser powder bed fusion. The results show that the TiC is uniformly distributed in the microstructure. With the addition of TiC, the average size of the grains is significantly reduced. The strength, hardness, and wear resistance of TiC/316L samples have been greatly improved. The tensile strength of formed 2 wt% TiC/316L is 948 MPa, together with a extension rate of 36.0%, which has been increased by 42.6% and 79.7%, respectively. This study provides an effective way to improve the strength at room temperature and the high temperature of 316L built by laser powder bed fusion.

## 1. Introduction

316L stainless steel owns the advantages of corrosion resistance, antioxidant properties, and cheapness, which has been widely used in industries such as chemical and nuclear engineering [1,2]. Laser powder bed fusion (LPBF) is a promising manufacturing process that has the advantages of a small melting zone and a fast cooling rate, which could help to avoid the segregation of the reinforcements [3]. Studies have shown some attempts of using LPBF to fabricate 316L composite parts [4].

However, the strength and wear resistance of 316L built by LPBF still cannot meet up the requirements of advanced applications. The microstructure and porosity of built parts have a strong influence on their mechanical performance. The variations of L-PBF parameters on microstructural characteristics and mechanical properties are still debatable [5]. Niendorf et al. found that a higher laser power promoted the induction of strong crystallographic textured columnar coarse-grains, which also significantly improved the ductility of L-PBFed 316L SS [6]. Maria et al. [7] stated that lower power facilitated the formation of a more random and finer cell-like microstructure in 316L fabricated by LPBF. Salmanm et al. stated that the scanning strategy significantly affected the characteristic size of cell-like structures and grains, as well as the tensile strength of L-PBFed 316L [8].

In terms of porosity, Pragana et al. [9] provided insight on the effect of operating parameters such as vector size and gas atmosphere (Nitrogen and Argon) on the part density. A negative influence of vector size was validated on the part density. A maximum relative density of 99.87% was achieved using a nitrogen atmosphere. Tobias et al. found that the shape, size, orientation, and distribution of pores are crucial parameters affecting mechanical properties [10].

Although efforts have been devoted to processing parameter optimization to improve microstructures, relative density, and tensile properties, the tensile strengths of LPBF-ed 316L parts were still lower than 800 MPa, which were 700 MPa [11], 751.6 MPa [12], 710 MPa [13], and 745 MPa [14]. At present, the introduction of secondary-phase particles in 316L to improve the intensity of materials has become an important approach [3,15]. Zhao et al. reported the fabrication of TiC/316L nanocomposites using LPBF [14]. However, the TiC/316L nanocomposite has a low ultimate tensile strength of 748.6 MPa, which is even lower than LPBF-fabricated 316L with no additions. Zhao et al. stated that adding 3 wt% micronsized and nanosized TiC particulates drastically refines the grains [14]. Besides, TiC particles can significantly strengthen 316L (from 609 MPa to 832 MPa) while maintaining the ductility at a high level (29%). However, present studies have not achieved the highest tensile strength of the TiC/316L composite. The mechanical performance at high temperatures for the TiC/316L composite is still unknown.

In this study, we intend to enhance the strength of 316L by the addition of a nano-sized TiC particle. In order to achieve a reliable result, we will optimize the process parameters of LPBF for achieving near full relative densities of 2 wt% TiC/316L and 4 wt% TiC/316L samples. With the analyses of element distributions, microstructure, tensile tests, and fractures, we will then discuss the strength enhancement of the addition of nano-sized TiC particles.

## 2. Materials and Methods

### 2.1. Materials Preparation

**Figure 1:**
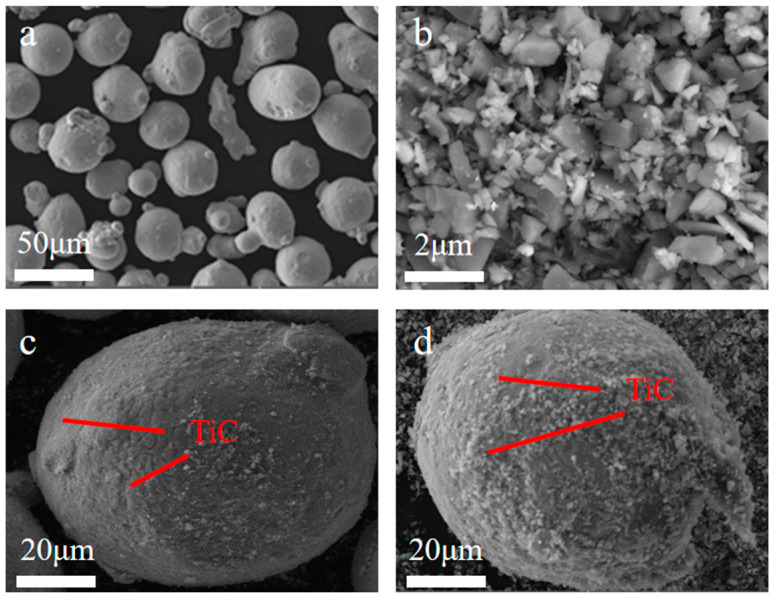
SEM images of powder and composite. (**a**) 316L; (**b**) TiC; (**c**) 2 wt% TiC/316L mixed powder; (**d**) 4 wt% TiC/316L mixed powder.

The 316L stainless steel powder used in this study was prepared by the gas atomization method, with a size range from 15 μm to 53 μm, as shown in Figure 1a. TiC powder was prepared by the self-propagating high temperature synthesis (SHS) method, with an average diameter of about 50 nm. In order to explore the impact of TiC powder content on the mechanical performance of the TiC/316L composite, the contents of the TiC powder were set to be 0 wt%, 2 wt%, and 4 wt%, respectively. The composite powder was produced by a ball milling method. The 316L powder, nano-sized TiC particle, and Zirconia ball (diameter of 5 mm) were mixed in a tank. With the high-speed rotation of the tank, the nano-sized TiC particle could be smashed and attached to the surface of the 316L powder. The optimized parameters of ball milling are shown in Table 1. The micromorphology of 2 wt% TiC/316L, 4 wt% TiC/316L composite powder is shown in Figure 1c,d. It can be seen from the figures that the nano TiC particles are evenly attached to the surface of the 316L particles.

### 2.2. Fabrication

**Figure 2:**
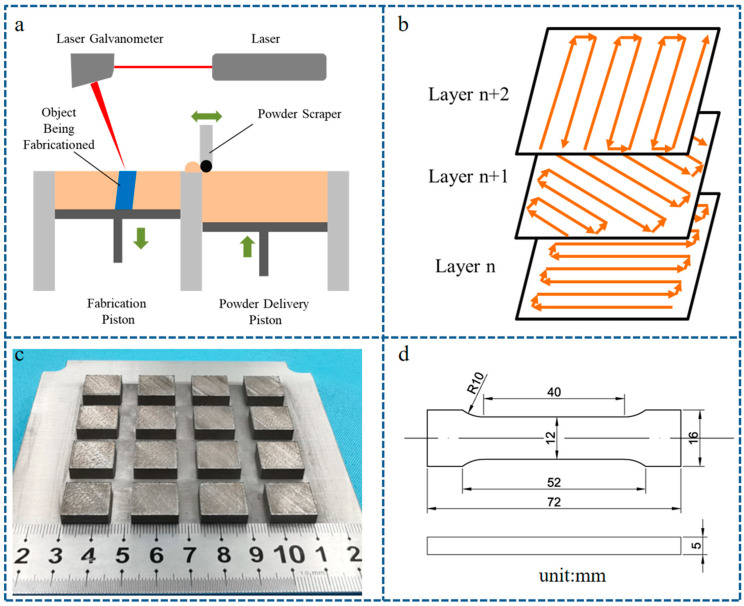
Laser powder bed fusion experiment. (**a**) Schematic diagram of LPBF; (**b**) scanning strategy; (**c**) optimization of processing parameters for TiC/316L composite; (**d**) schematic of tensile sample.

The laser powder bed fusion experiment was performed with an NCL-M2120 machine (Chamlion, Co., Ltd., Nanjing, China). It is equipped with a fiber laser (IPG, Co., Ltd., Boston, MA, USA) with a maximum power of 500 W. The wavelength of the laser is 1064 nm. The size of the focused laser spot is about 50 μm. The process of laser powder bed fusion is shown in Figure 2a, which is performed in an argon atmosphere. The meander scan strategy, together with a rotation of 67° every layer, is used, as shown in Figure 2b. Various parameters of laser power and scan speed were explored for each material, as shown in Figure 2c. The optimized processing parameters for each material are shown in Table 2.

### 2.3. Tests

The formed samples were cut from the substrate by wire electrical discharge machining (WEDM). The densities of the samples were tested based on the Archimedes drainage method. The cross-sections were then obtained by WEDM. They were etched by 5 g FeCl_3_ + 10 mL HCl + 10 mL H_2_O after being polished. An optical microscope (Olympus GX41, Tokyo, Japan) and scanning electron microscope (Hitachi S-4800, Tokyo, Japan) were used for microstructure observation. The distribution of elements was measured by an EDS energy spectrometer. Electronic back scattering diffraction (EBSD) with a pace of 0.30 μm was used to study the microstructure, crystal structure, and grain sizes of the sample. X-ray diffraction was performed with a D/MAX 2500VL/PC diffraction instrument (Rigaku, Tokyo, Japan). The microhardness of the samples was tested by using an HXS-1000 microhardness test instrument (Shangguang, Shanghai, China), with a load of 500 g and dewelling time of 18 s. The tensile property at room temperature of the fabricated sample (refer to ISO 6892-1: 2016 [16] for the room temperature test) was tested by an Instron 5982 universal tensile testing machine with a strain rate of 10^−3^/s. The tensile property at high temperature was tested at 300 °C, 500 °C, and 700 °C, respectively. It referred to ISO 6892-2: 2016 [17], which is used for high-temperature tensile tests. The test was performed by an Instron 5869 high-temperature tensile testing machine. The heating time of the samples was 20 min. The strain rate was set to be 10^−3^/s.

## 3. Results

### 3.1. Phase Analysis

**Figure 3:**
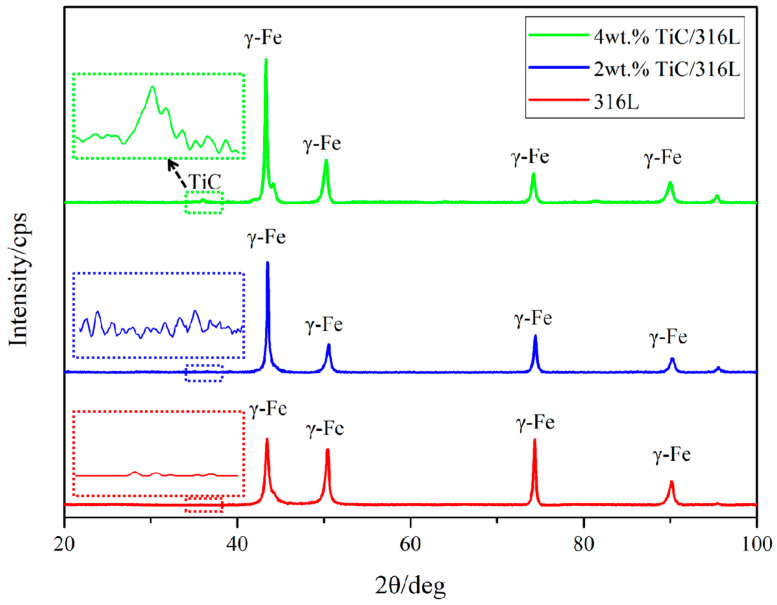
XRD spectrum of SLM-formed samples.

The X-ray diffraction results of 316L, 2 wt% TiC/316L, and 4 wt% TiC/316L are shown in Figure 3. It can be seen that all of the maximum diffraction peaks of the three samples are γ-Austenite (111). No TiC-related peak can be found in the sample of 316L. There are tiny peaks related to TiC in the sample of 2 wt% TiC/316L, which may due to the low content of TiC in the composite. There are small peaks related to TiC in the sample of 4 wt% TiC/316L. This shows that the TiC diffraction peak intensity increases with the content of TiC.

According to the Debye–Scherrer formula:

(1) $$D=\frac{K\lambda }{B\cos\theta },$$

where *D* is the average thickness of the crystal perpendicular to the direction of the crystal surface, *B* is the half-width of the sample diffraction peak. *K* represents the Scherrer constant. It can be deduced that the average size decreases with the increase of the half-width of the diffraction peak. As shown in Figure 3, the half-width of X-ray diffraction of the 4 wt% TiC/316L sample is the largest, while the half-width of the 316L sample is the least. Thus, with the increase of the addition of TiC, the half-width of the diffraction peak increases, indicating a decrease in grain size.

### 3.2. Microstructure

**Figure 4:**
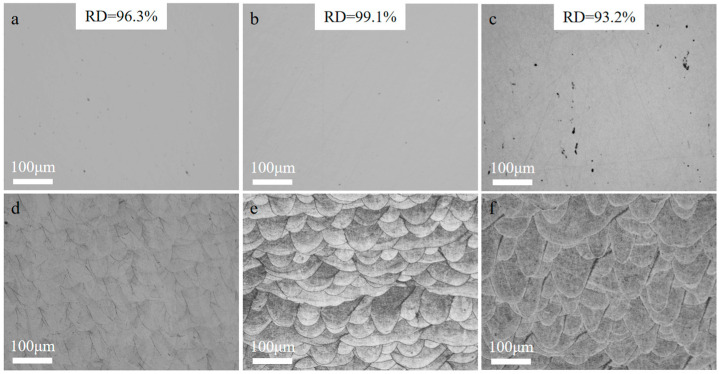
Optical microscope images of samples. (**a**) 316L before etched; (**b**) 2 wt% TiC/316L before etched; (**c**) 4 wt% TiC/316L before etched; (**d**) 316L after etched; (**e**) 2 wt% TiC/316L after etched; (**f**) 4 wt% TiC/316L after etched.

Figure 4 demonstrates the optical microscope images of the cross-sections of all samples. The optical images of the samples before etching reflect the relative densities to a certain extent. They are similar to the relative densities results tested by the Archimedes drainage method, which are 96.3% for 316L, 99.1% for 2 wt% TiC/316L, and 93.2% for 4 wt% TiC/316L, respectively. As shown in Figure 4d–f, the molten pools of all samples are like a fish scale. The molten pool of the 316L sample is narrow, while the molten pool of the 2 wt% TiC/316L sample is wider. The molten pool of the 4 wt% TiC/316L sample is wider and deeper. Nano-sized TiC particles may be beneficial to the higher absorption of laser energy, which leads to a wider and deeper molten pool.

**Figure 5:**
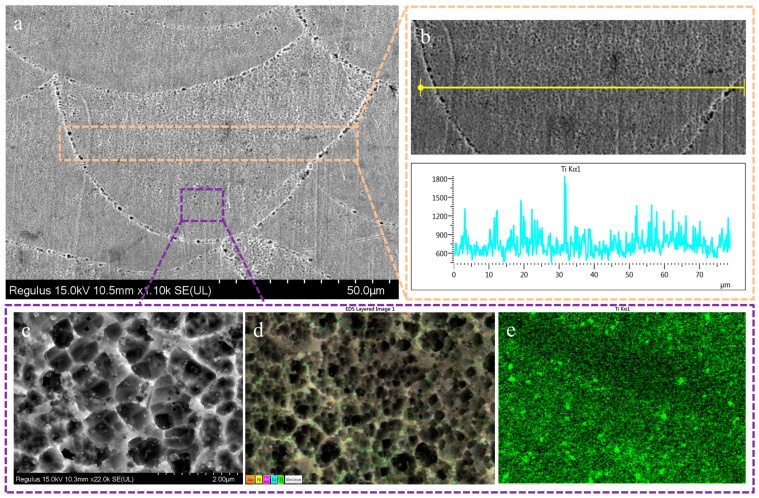
SEM and EDS analysis diagram of 2 wt% TiC/316L sample formed by SLM. (**a**) SEM side-view of formed sample; (**b**) EDS line scan of corresponding area; (**c**) SEM diagram of local area; (**d**) EDS diagram of local area; (**e**) Ti element distribution diagram of local area.

The element distribution of the cross-section of the 2 wt% TiC/316L sample are shown in Figure 5. The shape of the melting pool is clear. The size of the cell-like grains in the melting pool is uniform, as depicted in Figure 5a. Figure 5b shows that the Ti element is evenly distributed. It can be seen in Figure 5b that the X-ray energies at several points are relatively higher, which may be because larger-sized TiC particles (about 200 nm) were scanned. Figure 5c shows a raw image of the microstructure at the center of the melting pool. Figure 5d depicts the distribution of the main elements, including Mo, Ni, Fe, Cr, and Ti. As shown in Figure 5e, the EDS mapping results indicate that the TiC particles, which are depicted in bright green, are basically uniformly distributed around the cell crystal. There is a limited time for the growth of TiC nanoparticles, owing to the ultrahigh cooling rate (about 10^6^ K/s) of the LPBF process [18,19]. The 4 wt% TiC/316L sample composite has a similar microstructure and element distribution; thus, it is not shown here.

**Figure 6:**
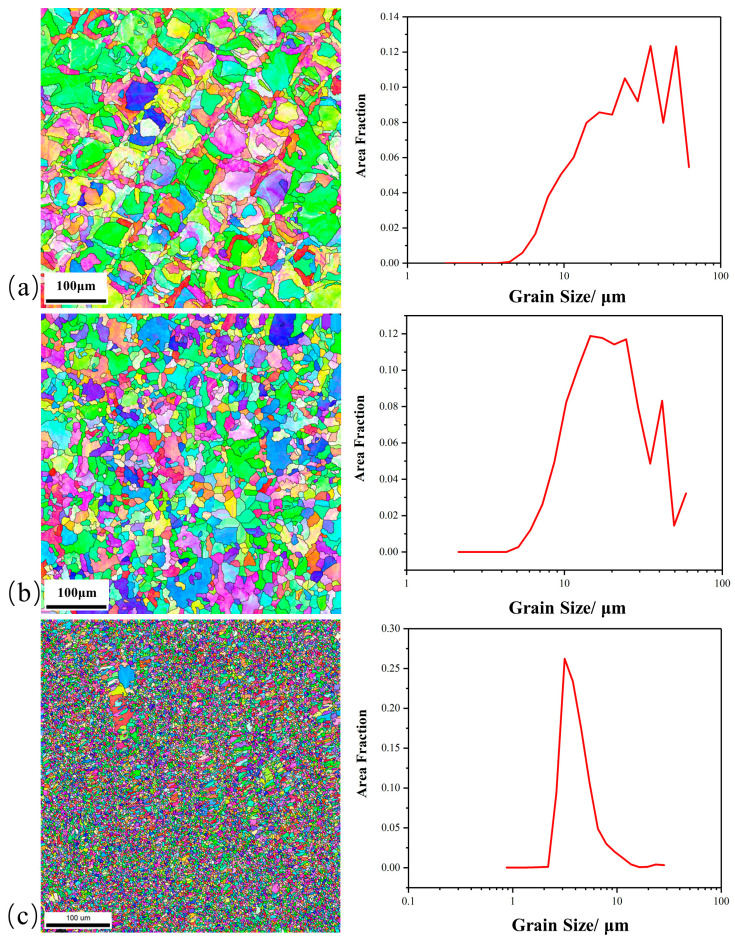
EBSD grain spectrum. (**a**) 316L; (**b**) 2 wt% TiC/316L; (**c**) 4 wt% TiC/316L.

Electronic back scattering scattering diffraction (EBSD) was used to measure the grain sizes of all samples. As shown in Figure 6, the average grain sizes of the 316L sample, 2 wt% TiC/316L sample, and 4 wt% TiC/316L sample are about 28.8763 μm, 21.8403 μm, and 4.51446 μm, respectively. The very fine grain obtained in the 4 wt% TiC/316L sample may be the finest grain size in the currently published literature about LPBF-ed 316L [20,21]. The TiC particles are beneficial to grain refinement, which may be attributed to the role of the crystalline core of the TiC particle in the molten pool. In the molten pool, austenitic grains nucleate on the surface of TiC particulates heterogeneously and, thus, a refined microstructure is obtained.

### 3.3. Microhardness

**Figure 7:**
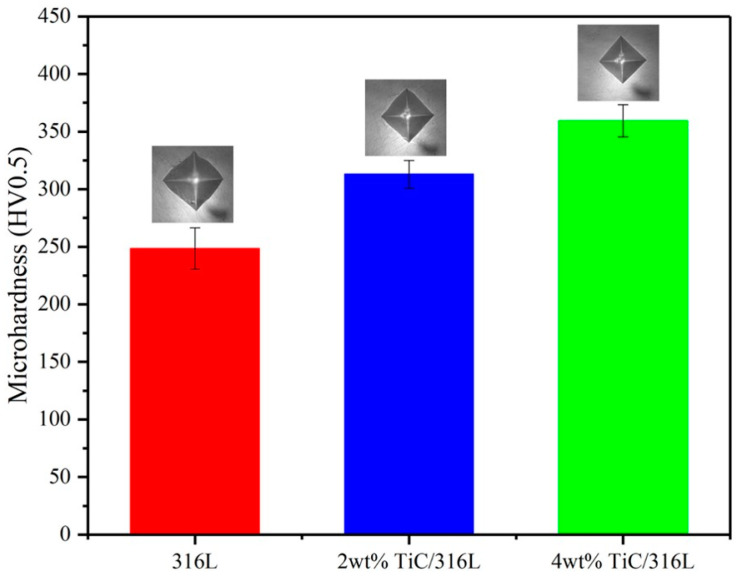
Microhardness values of all samples.

Figure 7 shows the microhardness values of the fabricated samples. It can be seen that the microhardness value gradually increases with the increase of TiC content. Compared with the microhardness of the 316L sample, the microhardness of the 2 wt% TiC/316L sample increased by about 25%. The microhardness of the 4 wt% TiC/316L sample increased by nearly 45%. The microhardness of the metal composite mainly depends on the content of the ceramic enhanced phase and the density of the composite sample [15]. In this study, we have explored optimized process parameters and have fabricated near full-density samples. At the same time, the existence nano-sized TiC also contributes significantly to the improvement of the microhardness of TIC/316L composite samples.

### 3.4. Tensile Property at Room Temperature

**Figure 8:**
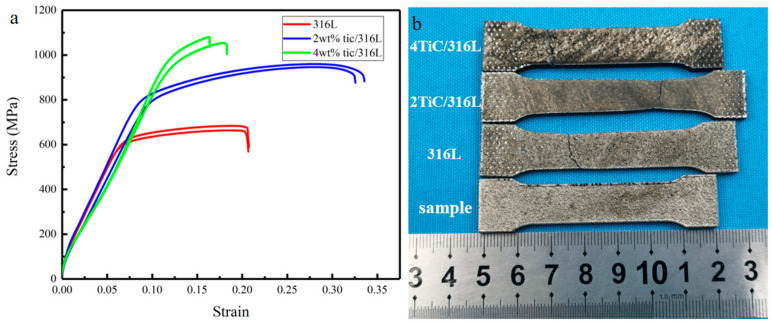
Tensile curves and samples after tensile test. (**a**) Tensile curves. (**b**) Samples after tensile test.

Figure 8 depicts the tensile properties of all samples at room temperature. The yield strength of the 316L sample is 563 MPa, while the tensile strength is 665 MPa. The yield strength of the 2 wt% TiC/316L sample is 789 MPa, while the tensile strength is 948 MPa, which increased by 42.6% and 40.2%, respectively. In addition, the ductility of the 2 wt% TiC/316L sample also obviously increased compared with that of the 316L sample. With the increase of TiC content, the yield strength of the 4 wt% TiC/316L sample is 960 MPa, while the tensile strength is 1080 MPa. However, its ductility decreased significantly. As stated by Stef et al., the presence of a pore defect makes the sample less ductile and more brittle [22]. Li et al. discovered that the tensile strength of the LPBF 316L sample decreases with an increase in porosity [23]. It can be deduced that the tensile strength and ductility of the 93% relative density sample may be lower than that of the 96% and 99% relative density samples. However, the tensile strength of the 4 wt% TiC/316L sample with 93% relative density is larger than that of the 316L sample with 96% relative density and the 2 wt% TiC/316L sample with 99% relative density. This means that the enhancement of the TiC particle plays a more significant role on the tensile strength of the sample than that of a pore defect. However, the ductility of the sample seems to be dominated by pore defects.

**Figure 9:**
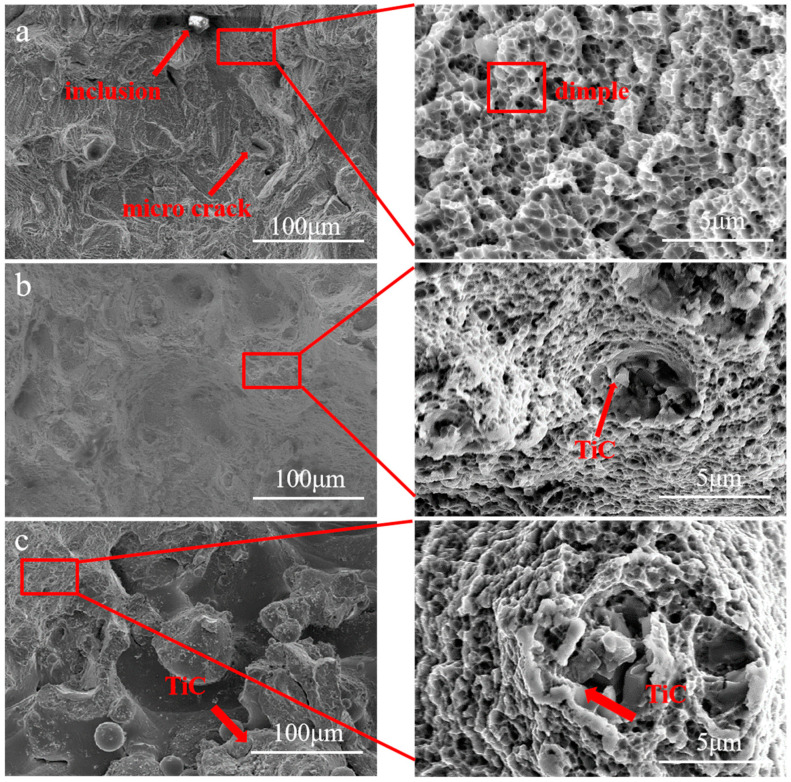
Tensile fracture morphology. (**a**) 316L; (**b**) 2 wt% TiC/316L; (**c**) 4 wt% TiC/316L.

The fracture microstructures are shown in Figure 9. There were many dimples at the fracture of the 316L sample, but there was no particle in it. The inclusion at the fracture shows that it may weaken the tensile property of the 316L sample. The microcracks at the fracture indicate that a large amount of tensile energy was exhausted by them. Many dimples can also be found at the fractures of the 2 wt% TiC/316L sample and 4 wt% TiC/316L sample. The presence of fine dimples indicates a ductile fracture mode. Many TiC particles can be found in the dimples. It should be noted that the TiC particle failed to fall from the dimple, which provides evidence of a tight combination of TiC particles and 316L. No microcracks were observed at the fracture of the 2 wt% TiC/316L sample and 4 wt% TiC/316L sample. This also indicates that the TiC particles directly bear the load during the tensile process, which greatly improves the tensile strength of the built part.

### 3.5. Tensile Property at High Temperature

**Figure 10:**
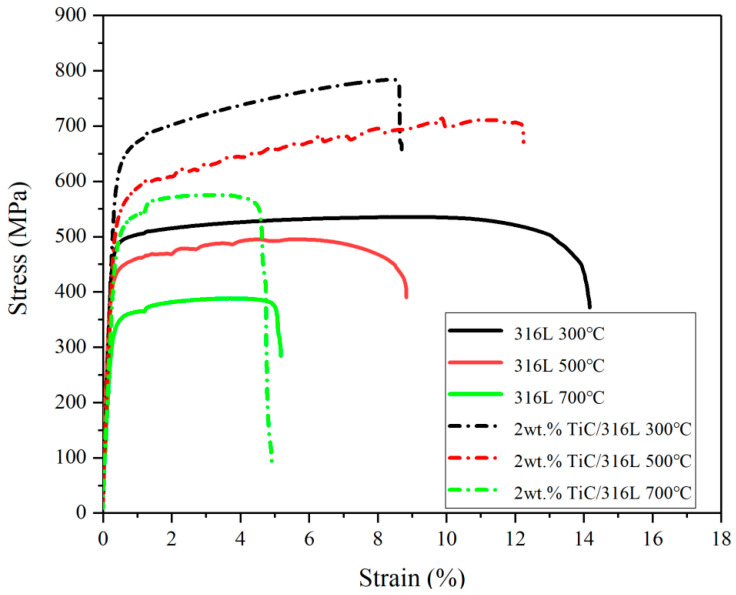
Tensile properties of 316L sample and 2 wt% TiC/316L sample at high temperature.

Figure 10 shows the tensile properties of the 316L sample and 2 wt% TiC/316L sample at high temperatures. It can be seen that the tensile properties of all samples decrease with an increase in test temperature. At the condition of 300 °C, the tensile strength of the 2 wt% TiC/316L sample is 785 MPa, which is 51% higher than that of the 316L sample. Even at the conditions of 500 °C and 700 °C, the tensile strengths of 2 wt% TiC/316L samples are much higher than that of 316L samples. The results indicate that the nano TiC particle can still play a significant role in strength enhancement at high temperature. On one hand, the hard TiC particle was pinned in the 316L substrate, which can effectively obstruct the deformation of 316L and crack propagation during tensile performance. On the other hand, the closely bonded TiC particle can bear a heavy load, resulting in an improvement of tensile performance. The above roles of the TiC particle are much more significant for tensile strength enhancement, especially when the 316L substrate is softened at high temperature.

## 4. Discussion

### 4.1. Strength Enhancement

Pore defects may have some influence on the mechanical properties of built parts. In this study, we have fabricated samples with the highest relative densities that we can achieve. The results have shown that nano-sized TiC attributes greatly to the improvement of 316L. The strengthening mechanism of nano TiC was analyzed according to the common strengthening mechanism of metal matrix composites. It is believed that the strength improvement is caused by the Orowan mechanism, fine grain reinforcement, and load transmission at the interface between nano TiC particles and the 316L matrix.

(1) Dispersion strengthening means that the second-phase particles are distributed in the alloy, which become effective obstacles of dislocation movement. The hardness of the TiC particle is far more than 316L. Their relationship is non-coherent. As such, the dislocation cannot pass through the TiC particle. The movement of dislocation will have to move via an Orowan round, overcoming the reaction force. The strength improvement can be illustrated by the Orowan formula [24]:
  (2) $$∆\sigma_{orowan}=\frac{\varphi G_{m}b}{d_{p}}(\frac{6V_{p}}{\pi }{)}^{\frac{1}{3}},$$
  where $G_{m}$ is the shear modulus of 316L (about 78 GPa); *b* is the Burgers vector, which is about 0.254 nm; $d_{p}$ and $V_{p}$ are the mean volume fraction and average size of TiC, which are 6.37 vol% and 50 nm, respectively; and φ is a constant, which is set to be 2.

According to this formula, the strength improvement of the Orowan mechanism is 157 MPa for 2 wt% TiC/316L and 245 MPa for 4 wt% TiC/316L. It should be stated that the shape and direction of the TiC particle were not taken into consideration, which may also influence the calculated results [25].

(2) For metal-base composite materials, the introduction secondary-phase particles will probably alter the crystalline dynamics of the molten pool. On one hand, some of the nano particles may become the seeds for the crystal. On the other hand, the rest of the nano particles may obstruct the growth of the grain and, thus, can form fine grains. As demonstrated in Figure 6, the average sizes of the grains decreases significantly with the increased content of the TiC addition. According to the Hall–Petch relationship, the improvement of strength caused by grain refinement can be calculated as follows:
  (3) $$\Delta \sigma_{H-P}=k\cdot d^{-\frac{1}{2}},$$
  where *k* is the Hall–Petch constant, which is set to be 17.4 $\mathrm{MPa}\times {\mathrm{mm}}^{1/2}$. Thus, the strength improvements will be 117.85 MPa for 2 wt% TiC/316L and 259.1 MPa for 4 wt% TiC/316L.
(3) The main enhancement mechanism of particle-enhanced composite materials is the transfer of the load from the matrix to the high-strength particle [26]. In other words, the high-strength particles undertake much of the load. As can be seen from Figure 9, the TiC particles did not fall of from the matrix of 316L. Based on the tight bonding between TiC and 316L, the nano TiC particles may undertake much of the load. The strength improvement caused by bonding can be calculated as follows:
  (4) $$\Delta \sigma_{LT}=\frac{1}{2}V_{p}\sigma_{m},$$
  where $\sigma_{m}$ is the tensile strength of 316L. Thus, the strength improvements caused by bonding will be 110.59 MPa for 2 wt% TiC/316L and 21.18 MPa for 4 wt% TiC/316L.

In all, the improvement of strength can be calculated as follows:

(5) $$\Delta \sigma =\Delta \sigma_{orowan}+\Delta \sigma_{H-P}+\Delta \sigma_{LT}.$$

Based on the above formula, the improvements of strength are 285.44 MPa for 2 wt% TiC/316L and 525.28 MPa for 4 wt% TiC/316L. The calculated strength improvement of the 2 wt% TiC/316L sample is quite similar to the experimental result, i.e., 283 MPa. However, the calculated strength improvement of the 4 wt% TiC/316L sample is a little larger than the experimental result, i.e., 415 MPa. It can be deduced that many more TiC particles are prone to form a cluster (as shown in Figure 9c), which is easy to be initially cracked. In addition, the relative density of the 4 wt% TiC/316L sample is about 93%. The inner pore defect may be detrimental to the tensile strength [23].

### 4.2. Ductility Improvement

In most cases, an improvement of metal strength is usually established on the sacrifice of ductility. An interesting finding in this study is that the ductility and strength of the 2 wt% TiC/316L sample are both improved. Herein, we will try to make an interpretation.

Fine grains are formed with the addition of the nano TiC particle. The finer the grain size of the alloy material, the higher the plasticity of the material. This can be attributed to the decrease of stress concentration and more even plastic deformation for metal with fine grains [27]. Finer grains of metal mean a more uniform distribution of grains; thus, the load between the grains is also uniform, which is beneficial to reduce the degree of stress concentration and the probability of cracking. Furthermore, the plastic deformation stage of the material is relatively stable and uniform [28].

## 5. Conclusions

In this study, a TiC/316L composite was prepared by LPBF. The strength enhancement has been discussed. Several conclusions can be drawn as follows.

(1) The 2 wt% TiC/316L sample with near full density was prepared by optimizing the process parameters in LPBF.
(2) The average sizes of grains in the TiC/316L composite decreased with the increased content of TiC.
(3) The tensile properties at both room temperature and at high temperature for the 2 wt% TiC/316L sample were significantly enhanced.
(4) The strength enhancement may attributed to the Orowan mechanism, fine grain strengthening, and load-transferred strengthening.

## Figures and Tables

**Table 1:** Parameters for ball milling.

Content (wt%)	Time (h)	Speed (rpm)	Milling Time (min)	Dwelling Time (min)
2	4	120	20	20
4	8	150	20	20

**Table 2:** Optimized processing parameters for each material.

Content (wt%)	Laser Power (W)	Scan Speed (mm/s)	Layer Thickness (μm)	Hatch Distance (μm)
0	180	1000	30	60
2	200	1000	30	60
4	240	800	30	60

## Data Availability

Data are contained within the article.
